# Phospholipid flippase ATP11A brokers uterine epithelial integrity and function

**DOI:** 10.1073/pnas.2420617122

**Published:** 2025-04-22

**Authors:** Alexa Krala, Aleksandra O. Tsolova, Bethany N. Radford, Anshul S. Jadli, Xiang Zhao, Danielle Blackwell, Ankita Narang, Wendy Dean, Myriam Hemberger

**Affiliations:** ^a^Department of Biochemistry and Molecular Biology, Cumming School of Medicine, University of Calgary, Calgary, AB T2N 4N1, Canada; ^b^Alberta Children’s Hospital Research Institute, University of Calgary, Calgary, AB T2N 4N1, Canada; ^c^Department of Cell Biology and Anatomy, Cumming School of Medicine, University of Calgary, Calgary, AB T2N 4N1, Canada

**Keywords:** uterus, development, placenta, heart, cell membrane

## Abstract

Uterine adaptations driven by the steroid hormones estrogen and progesterone are pivotal for embryo implantation and, ultimately, for a successful pregnancy. Here, we show in mice that genetic ablation of the membrane lipid flippase *Atp11a* causes severe deficits in this hormonal response and profound defects in the morphological organization and transcriptional profile of the uterine epithelial compartment where *Atp11a* is expressed. *Atp11a*-null uterine epithelial cells lack tight junctions, and the luminal epithelium exhibits profound disruptions to cellular morphology. Interestingly, the specification of luminal epithelial cells remains incomplete as they maintain expression of the normally gland-restricted marker FOXA2. The uterine glands of *Atp11a*-null females are depleted for progenitor cells marked by SOX9, PAX8, LGR5, and PROM1. Collectively, these findings point to a uterine receptivity deficit that underpins the frequent failure of *Atp11a*-depleted females to establish a successful pregnancy. Most intriguingly, however, loss of only a single functional *Atp11a* allele causes a higher frequency of abnormal placental trophoblast differentiation as well as a higher incidence of developmental heart defects in wild-type embryos. These data emphasize the far-reaching impact of uterine dysfunction on reproductive outcome and highlight the importance of the maternal genotype in the etiology of developmental disorders.

## Significance

In the current study, the authors describe severe uterine defects in female mice depleted in the phospholipid flippase ATP11A, highlighting the importance of membrane biology and lipid bilayer composition for uterine function. *Atp11a* deficiency increases the rate of implantation failure by 50% due to profound structural abnormalities of the uterine luminal epithelium. Importantly, although the remaining 50% of females carry litters of normal size, their placentas are transcriptionally compromised and the embryos exhibit a greater risk of developmental heart defects. These data demonstrate that uterine gland deficiencies impact placental development which in turn imposes a greater risk of developmental disorders even in genetically normal embryos.

Embryo implantation is a milestone event that sets the stage for a successful pregnancy. The uterus undergoes dramatic morphological and molecular changes that are primarily driven by the steroid hormones estrogen and progesterone (P4) to reach a receptive state ready for blastocyst attachment and implantation (1, 2). In mice, preovulatory estrogen triggers an extensive amount of proliferation in uterine epithelial cells. Rising levels of P4 derived from the corpus lutea superimposed onto preimplantation ovarian estrogen secretion become critical for uterine receptivity at embryonic day (E)3.5, inducing a shift of proliferation from the epithelial compartment to the endometrial stromal cells. Concomitant with its antiproliferative effect on the epithelium, P4 action induces epithelial cell differentiation and glandular secretions. P4 also drives key transformations to the uterine stroma by inducing the decidualization reaction. Due to these vital roles, P4 is an absolute requirement for implantation in all species studied (1, 2).

In mice, uterine glands develop after birth and derive from cells of the luminal epithelium (3). Thus, the glandular epithelium (GE) and the luminal epithelium (LE) are continuous, even if the glands commonly appear as circular structures on histological sections (4). Despite this common origin, the expression signatures of GE and LE cells diverge, and so does their subcellular organization. LE cells are characterized by a microvillous apical surface positive for acetylated tubulin, whereas the GE is demarcated by the expression of several key transcription factors including FOXA2 and SOX9 that are restricted in their expression to the GE (5, 6). Additional gene expression differences have been uncovered using laser microdissection experiments of GE and LE that revealed distinct expression signatures defining both epithelial compartments (7).

All three main components of the mouse uterus, i.e., the LE, GE, and uterine stroma, are critical for a successful pregnancy and rely on continuous crosstalk. The LE of the receptive uterus provides the surface for direct interaction with the blastocyst, a process that is facilitated by the presence of various glycosylated proteins on its apical surface. These are recognized by carbohydrate-binding cell adhesion proteins expressed by the outer trophectoderm cells of the blastocyst. A prime example for such a protein in humans is L-selectin that facilitates blastocyst attachment; when blocked, the adhesion of trophoblasts to the endometrial epithelium is impaired (8–10). Other proteins, such as the polymeric and highly glycosylated MUC1, can mask the exposure of adhesive glycoproteins, and therefore, MUC1 is down-regulated in LE cells at sites of blastocyst attachment (10). As such, the organization and adequate hormonally mediated adaptation of the LE is paramount for uterine receptivity.

The critical role of uterine glands is evidenced by the complete infertility of glandless mice. This scenario can be achieved by P4 treatment of neonatal mice (11) or genetically by knockout (KO) of the transcription factor *Foxa2* or the cytokine *Wnt7a* (12–15). Collectively, these studies have established the vital role of uterine glands and their secretions for blastocyst implantation and stromal cell decidualization. Although females lacking uterine glands are generally infertile, the conditional *Foxa2* deletion in uterine glands can be temporarily compensated by LIF supplementation. While this treatment allows for implantation to occur, LIF-supplemented *Foxa2*-deficient glandless mice still exhibit decidualization defects leading to an abrupt loss of pregnancy at E7.5. This example demonstrates the importance of the crosstalk between uterine glands and the surrounding uterine stroma for the establishment and maintenance of pregnancy (16).

A failure in stromal cell decidualization also results in female infertility, although pregnancy loss occurs slightly later in the immediate days after implantation. This is exemplified by a uterine-specific KO of *Bmp2* in which the uterine stroma is incapable of undergoing the decidual reaction to support further embryonic development (17).

The vast majority of gene KO studies in the context of female reproductive tract function have assessed the roles of transcription factors, hormones, growth factors, and intracellular signaling components that guide embryo implantation and stromal cell decidualization. The importance of cell membrane composition for the reception and transmission of hormonal and growth factor signaling cues is less well known. Here, we follow up on observations of reduced pregnancy success in mice ablated for the phospholipid flippase *Atp11a* (18). ATP11A is a P4-type ATPase that functions at the plasma membrane to translocate phosphatidylserine (PtdSer) from the outer to the inner leaflet. Thereby, ATP11A helps maintain the disequilibrium of lipids across the plasma membrane where PtdSer and phosphatidylethanolamine are enriched on the inner leaflet, while phosphatidylcholine and sphingomyelin are exposed on the cell surface. This plasma membrane asymmetry is involved in many pivotal processes such as the regulation of membrane curvature, cell adhesion, cell fusion, and apoptosis (19, 20). We find that *Atp11a*-deficient female mice exhibit significant disruptions to the epithelial integrity of the uterine LE and GE cells, with a disorganized morphology and reduced expression of cell adhesion molecules. We also find a stark reduction in expression of critical GE progenitor cell markers, notably SOX9, and an apparent misspecification of some LE cells that retain GE marker signatures. Expression profiling reveals a specific failure of hormone-responsive genes to become activated to appropriate levels. Collectively, these deficits serve to account for the 50% failure rate of *Atp11a*-depleted females to establish a successful pregnancy and implicate membrane phospholipid biology in uterine function.

## Results

### Female *Atp11a* Deficiency Is Associated with Reduced Pregnancy Success Rates.

**Fig. 1.**
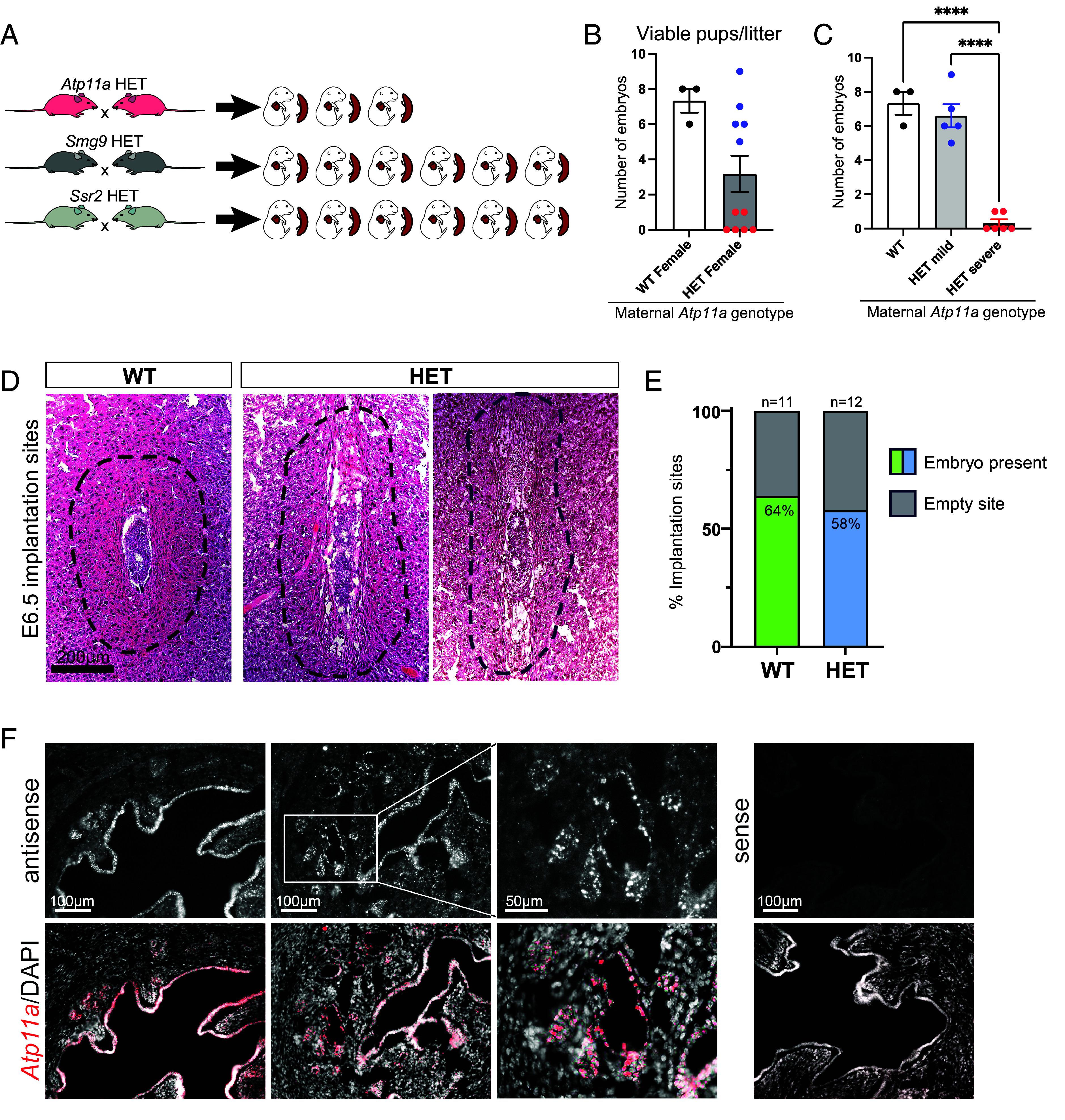
Reproductive performance of *Atp11a*^+/−^ females. (*A*) Diagram of the original observations on fewer embryos retrieved to crosses involving females HET for *Atp11a*, bred as part of a larger effort involving other mouse lines gene-targeted for *Smg9* and *Ssr2* that were selected for a placenta–heart focused project design (18). (*B*) Number of viable pups retrieved at E14.5 to WT and *Atp11a* HET females. Data are plotted as mean ± SEM. (*C*) Data as in *B* but with HET females split into those that contained only blood-filled uteri with no or one remaining pup (“severe”) versus other HET females that carried normal litters. Data are plotted as mean ± SEM. Statistical significance was calculated using one-way ANOVA. ****P < 0.0001. (*D*) H&E stainings of E6.5 implantation sites of WT and HET females, showing largely normal signs of decidualization and embryo development. The dashed line demarcates the primary decidualization zone. (*E*) Scoring of E6.5 implantation sites for the presence or absence of a normally implanted embryo. (*F*) In situ hybridization for *Atp11a* showing expression in the uterine epithelial compartments, i.e., in the LE and GE.

In previous breeding efforts centered around our interest in the placenta–heart axis, we had observed that females carrying a heterozygous (HET) gene deletion for *Atp11a* often failed to produce the same number of pregnancies compared to other mouse lines heterozygous for unrelated gene deletions, *Smg9* and *Ssr2*, that were bred in parallel (Fig. 1*A*) (18). This was despite the presence of a vaginal plug, indicative of a successful mating. To assess this anecdotal observation in more detail, we set up 11 *Atp11a* HET females with wild-type (WT) C57BL/6 males and dissected plugged females at embryonic day (E)14.5. In parallel, control matings were also set up with C57BL/6 WT females. Akin to our prior observations, we found that only around half of the *Atp11a* HET females (5/11) carried a litter in the normal size range of 6 to 9 pups. By contrast, 6/11 HET females had swollen, often blood-filled uteri, with either no viable pup or 1 single pup only (Fig. 1*B*). Because of this bimodal distribution of successful versus unsuccessful pregnancies (Fig. 1*C*), we tested for potential genomic imprinting or sex-specific effects in reciprocal (WT × HET) and (HET × WT) (female always named first) matings, but could not detect any parent-of-origin-dependent effect or sex-specific differences among the offspring (*SI Appendix*, Fig. S1 *A*–*E*). We also investigated E6.5 implantation sites of WT and HET females histologically, but although the tissue in HET females appeared less cell-dense, we did not observe major differences in embryo development (Fig. 1 *D* and *E*). Further confirmation of the principal ability to carry pregnancies to term was obtained by assessing litter sizes of *Atp11a* knockout (KO) females, generated by rescuing the embryonic lethality of the constitutive *Atp11a* KO by providing the null embryo with a functional placenta (18, 21). Average litter sizes of KO females were indistinguishable from those of WT females (*SI Appendix*, Fig. S1*F*). The successful formation of corpora lutea in KO females also indicated that they ovulated normally (*SI Appendix*, Fig. S1*G*). Thus, *Atp11a* deficiency appears to increase the risk of complete pregnancy losses, while females in which this early bottleneck is overcome can carry pregnancies to term.

### Transcriptional Profiling of *Atp11a*-Deficient Uteri.

**Fig. 2.**
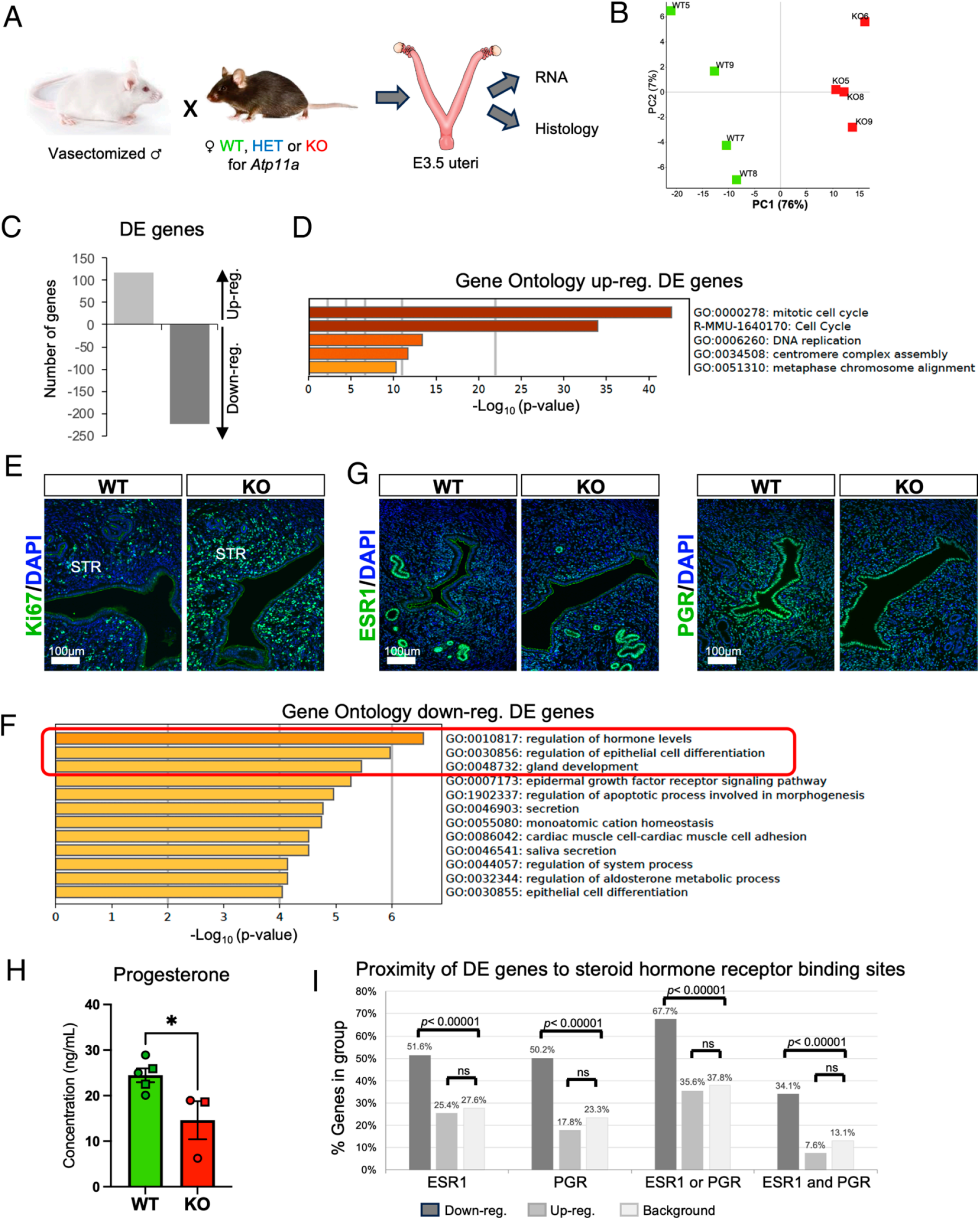
*Atp11a* KO uteri fail to activate hormonally regulated genes. (*A*) Experimental design for the retrieval of E3.5 primed uteri as a result of mating females of the indicated genotypes (WT = wild-type, HET = heterozygous, KO = knockout for *Atp11a*) to vasectomized males, which primes the uterus for implantation and produces a state of pseudopregnancy. (*B*) PCA on the entire transcriptomes of WT and KO uteri, with samples selected based on overall stage- and tissue homogeneity (*SI Appendix*, Fig. S2). (*C*) Number of up- and down-regulated differentially expressed (DE) genes as determined by DESeq2 analysis. (*D*) Gene Ontology analysis based on Metascape (23) for the up-regulated DE genes. (*E*) Immunofluorescence staining for the proliferation marker Ki67 shows higher proliferation rates in the uterine stroma of KO uteri. The absence of Ki67-positive cells from the LE of WT and KO uteri indicates correct hormonal priming. (*F*) Gene Ontology analysis based on Metascape (23) for the down-regulated DE genes. (*G*) Immunofluorescence staining for estrogen receptor (ESR1) and progesterone receptor (PGR) reveals similar expression patterns and intensities between WT and KO uteri. (*H*) ELISA for circulating progesterone (P4) levels in plasma from nonpregnant (circle) or E6.5 pregnant (square) WT and KO females. Pregnancy state did not interfere with the overall observation of reduced P4 levels in KO females. Statistical analysis was performed using the two-tailed unpaired *t*-test. *P < 0.05. (*I*) Proximity of DE genes to ESR1 and PGR genomic binding sites compared to genomic background. Proximity cutoff was set to 5 kb of the gene. The down-regulated DE genes are strongly enriched for ESR1 and PGR binding sites. Statistical analysis was performed with Fisher’s exact test.

To gain insights into the role of *Atp11a* in uterine receptivity and function, we performed in situ hybridization and detected *Atp11a* transcripts specifically in the epithelial compartment of the mouse uterus in LE and GE cells (Fig. 1*F*). We then performed RNA-seq on uteri of adult mice during the window of receptivity at E3.5 of pseudopregnancy as the result of matings to vasectomized males (Fig. 2*A*). To this end, we used 9 WT, 5 HET, and 9 KO females to dissect their uteri, cutting each horn into two to three pieces, which allowed us to perform RNA isolation and histological assessments on the same specimens (Fig. 2*A*). The genotype of these uteri was confirmed by inspecting RNA-seq reads over the targeted exons 7 and 8 of the *Atp11a* gene locus that were completely absent in all 9 KO samples (*SI Appendix*, Fig. S2*A*). Using initial sample similarity analysis tools based on Euclidean distances or principal component analysis (PCA) on all genes, no clear-cut separation based on genotype could be made out (*SI Appendix*, Fig. S2 *B* and *C*). Therefore, we interrogated our transcriptomic profiles more closely for developmental stage- and cell-type-specific expression signatures (7, 22), and found that the variance contributing to the two main principal components was introduced by subtle differences in the precise staging and cell type composition of our samples, independent of genotype (*SI Appendix*, Fig. S2 *D*–*G*). Consequently, we moved from an unsupervised to a supervised approach by selecting the samples (n = 4 WT and KO each) with the most similar expression signatures (*SI Appendix*, Fig. S2*G*), allowing for a more informed assessment of the impact of *Atp11a* deficiency while ruling out developmental staging-related variability. Using these eight developmentally stage-matched samples for a stand-alone analysis, they clearly separated into a WT and a KO group along PC1 which accounted for 76% of transcriptional variability (Fig. 2*B*).

### *Atp11a* Deficiency Preferentially Affects Hormonally Regulated Genes.

DESeq2 analysis on this subset of samples identified 341 DE genes, 118 of which were up-regulated and almost twice as many, 223 genes, were down-regulated (Fig. 2*C*). Further analysis of these DE genes revealed that the extent of reduced expression in KOs was far more pronounced than the degree of upregulation (*SI Appendix*, Fig. S3 *A* and *B*). Thus, the mean expression difference of the down-regulated genes was 1.5-fold lower, while that of the up-regulated genes was only 0.76 times higher in the KOs compared to WT control (*SI Appendix*, Fig. S3*A*). This finding might indicate that the down-regulated gene set is biologically more meaningful than the up-regulated genes, as the latter are subject to only a relatively modest transcriptional change.

Gene ontology analysis revealed that the up-regulated genes were enriched for cell cycle-related terms (Fig. 2*D*). Indeed, Ki67 staining revealed higher proliferation rates of stromal cells in the KO, which was somewhat surprising as the tissue is not larger in size (Fig. 2*E*). Importantly, the Ki67 staining pattern also confirmed that proliferation had correctly shifted from the epithelial into the stromal cell compartment in both WT and KO samples, as expected for E3.5 peri-implantation stage uteri (Fig. 2*E*) (24). The down-regulated genes were associated with the regulation of hormone levels, of epithelial differentiation, and with gland development (Fig. 2*F*), terms that are immediately pertinent to uterine receptivity and function. Prompted by these enrichment terms, we first stained the uteri for ESR1 and P4 receptor (PGR) but found no overt differences in these steroid receptor expression patterns between WT and KO samples (Fig. 2*G*). However, determining circulating hormone levels in the females revealed a significant reduction in the concentration of P4 in KOs and a great interindividual range in HETs, while estradiol levels remained unchanged (Fig. 2*H* and *SI Appendix*, Fig. S3*C*). Of note, P4 levels were even reduced in the KO female that had clearly ovulated (*SI Appendix*, Fig. S1*G*). Moreover, when interrogating the DE gene lists for proximity to ESR1 and PGR binding sites (25, 26), we found a strong enrichment for the down-regulated genes, two-thirds of which were close to either an ESR1- or PGR-bound site (Fig. 2*I*). By contrast, the relationship of up-regulated DE genes to ESR1/PGR-bound sites was similar to the genomic background (Fig. 2*I*). These findings demonstrate that *Atp11a*-deficient females exhibit reduced P4 levels resulting in reduced expression levels of steroid hormone-responsive genes, in particular those regulated by P4 (*SI Appendix*, Fig. S3*D*).

### Glandular Dysfunction in *Atp11a* KO Uteri.

**Fig. 3.**
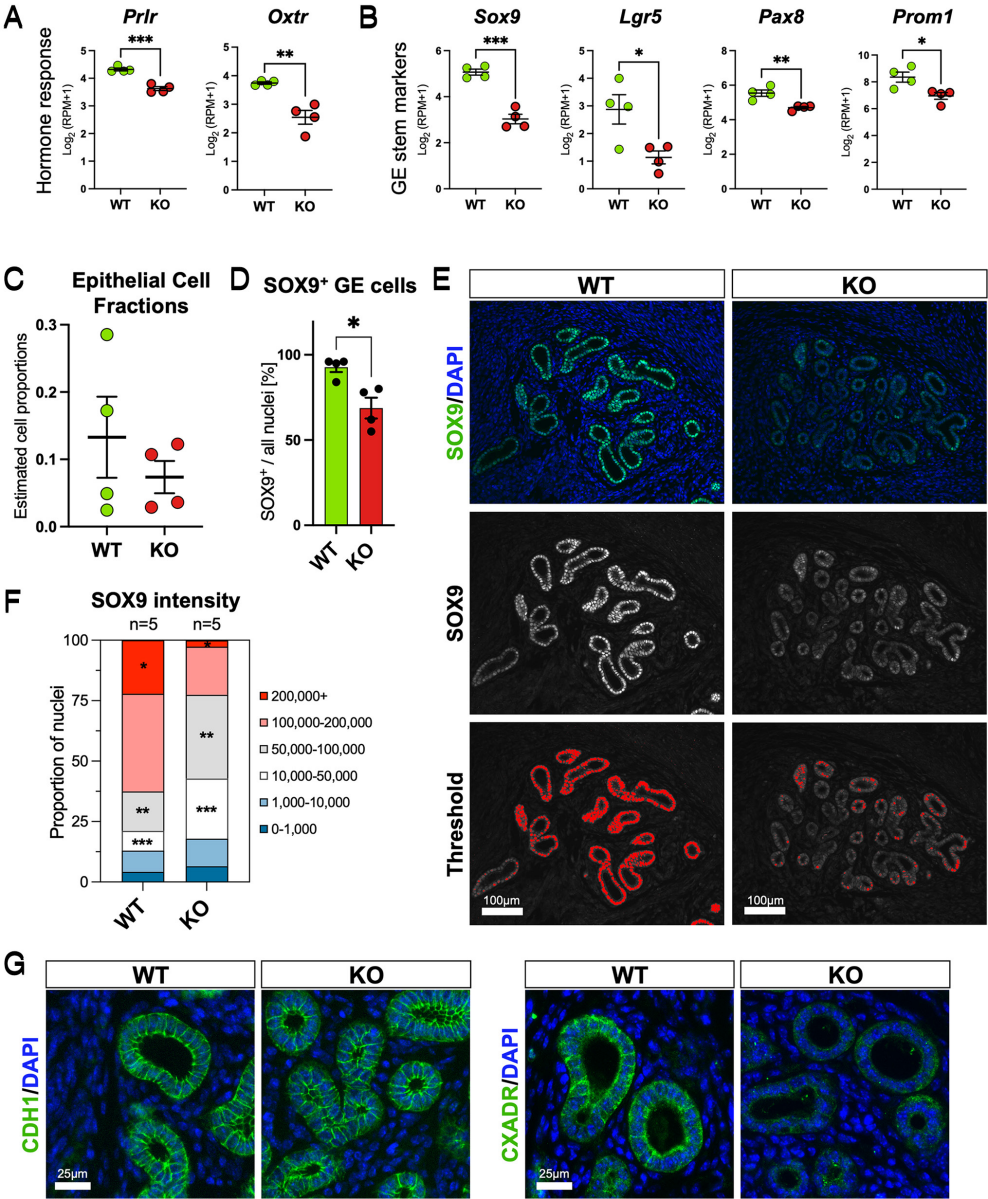
Glandular defects in *Atp11a* KO uteri. (*A*) Expression of *Prlr* and *Oxtr* as hormonally regulated genes. Log_2_(RPM + 1) values are displayed as mean ± S.E.M. Statistical analysis was performed using the two-tailed unpaired *t*-test. **P < 0.01; ***P < 0.001. (*B*) Expression of glandular stem/progenitor cell markers. Log_2_(RPM + 1) values are displayed as mean ± S.E.M. Statistical analysis was performed using the two-tailed unpaired *t*-test. *P < 0.05; **P < 0.01; ***P < 0.001. (*C*) Estimated proportions of epithelial cells in our bulk RNA-seq data based on single-cell sequencing data (29) using bisque cell-type-specific deconvolution analysis (30). (*D*) Quantification of SOX9-positive GE cells based on thresholding of immunofluorescence stainings shown in *E*. Data are displayed as mean ± SEM. Statistical significance was calculated using the Mann–Whitney *U* test. *P < 0.05. (*E*) Immunofluorescence staining for the glandular progenitor cell marker SOX9. The *Middle* row shows the black and white image of SOX9 staining that was used for arbitrary cutoff thresholding (*Lower* row) in ImageJ to determine positive and negative cells. (*F*) Quantification of fluorescence intensities in individual nuclei of uterine glands in WT and KO samples. The lower-intensity groups are enriched whereas the high-intensity groups are depleted in the KO uteri. Statistical significance was calculated using the Mann–Whitney *U* test. *P < 0.05; **P < 0.01; ***P < 0.001. (*G*) Immunofluorescence staining of uterine glands for the epithelial marker E-Cadherin (CDH1) and for the tight junction marker CXADR. CXADR is barely detectable in GE cells of KO uteri.

We then investigated whether the stark enrichment of steroid hormone binding sites at down-regulated DE genes (Fig. 2*I*) was functionally meaningful. Indeed, we observed a pronounced effect of *Atp11a* deficiency on the expression of genes that are under hormonal regulation, such as the prolactin and oxytocin receptors (*Prlr* and *Oxtr*) (Fig. 3*A*). Intersecting a list of genes that are strongly hormonally responsive in endometrial epithelial cells in vitro with the DE gene list of *Atp11a* KO uteri further revealed that principally all of the overlapping hormonally regulated DE genes were down-regulated in KO uteri (*SI Appendix*, Fig. S3*E*) (27, 28); in other words, they failed to become adequately activated. Thus, *Atp11a* KO uteri exhibited profound deficiencies in their transcriptional response to the pregnancy hormones that orchestrate the uterine changes required for receptivity and implantation.

Inspection of the down-regulated DE gene list highlighted several factors with known critical functions in the GE, notably *Sox9*, *Lgr5*, *Pax8,* and *Prom1* (Fig. 3*B*) (6, 27, 31). These factors have been specifically associated with a GE progenitor state displaying more stem cell–like characteristics and capable of regenerating the endometrial epithelium (6, 27, 31–34). Yet *Foxa2*, a transcription factor required for the establishment of uterine glands, was not differentially expressed in *Atp11a* KO uteri (*SI Appendix*, Fig. S3*F*). Similarly, other epithelial hallmark genes such as E-Cadherin (*Cdh1*), *Epcam,* or Keratin 18 (*Krt18*), and also the microvillous stabilizing factor ezrin (*Ezr*), remained unchanged, collectively suggesting that the expression differences were not caused by shifts in the proportional contributions of LE to GE or stromal cells (*SI Appendix*, Fig. S3*F*). These observations were in line with the histologically similar appearance of WT and KO uteri (*SI Appendix*, Fig. S3*G*). We investigated this point in further detail by performing a cell-type-specific deconvolution analysis on our bulk RNA-seq data using the bisque script (30), after carefully assigning cell-type-identifying gene sets based on recent single-cell RNA-seq data (29). This analysis confirmed the absence of significant changes in the proportions of the various cell types between WT and KO uteri (Fig. 3*C* and *SI Appendix*, Fig. S4*A*).

Because of the prominent reduction in transcript levels of multiple GE progenitor cell markers, we followed up on these findings with immunofluorescence stainings on sections of the identical uteri used for RNA-seq. Indeed, we observed significantly reduced staining intensities for the transcription factors SOX9 and PAX8 in uterine glands of *Atp11a* KO females (Fig. 3 *D* and *E* and *SI Appendix*, Fig. S4*B*). For an even more accurate assessment, we determined the range of immunofluorescence intensities in individual GE nuclei and observed significantly fewer brightly stained cells, whereas the number of cells with lower staining intensity was enriched (Fig. 3*F*). Finally, we inspected gland morphology more carefully and while we could not detect obvious differences in E-Cadherin (CDH1) staining, the tight junction marker CXADR was drastically reduced (Fig. 3*G* and *SI Appendix*, Fig. S3*F*), indicating that the membrane-localized ATP11A protein affects epithelial integrity.

### Uterine Epithelial Organoids Exhibit Unchanged Characteristics In Vitro.

**Fig. 4.**
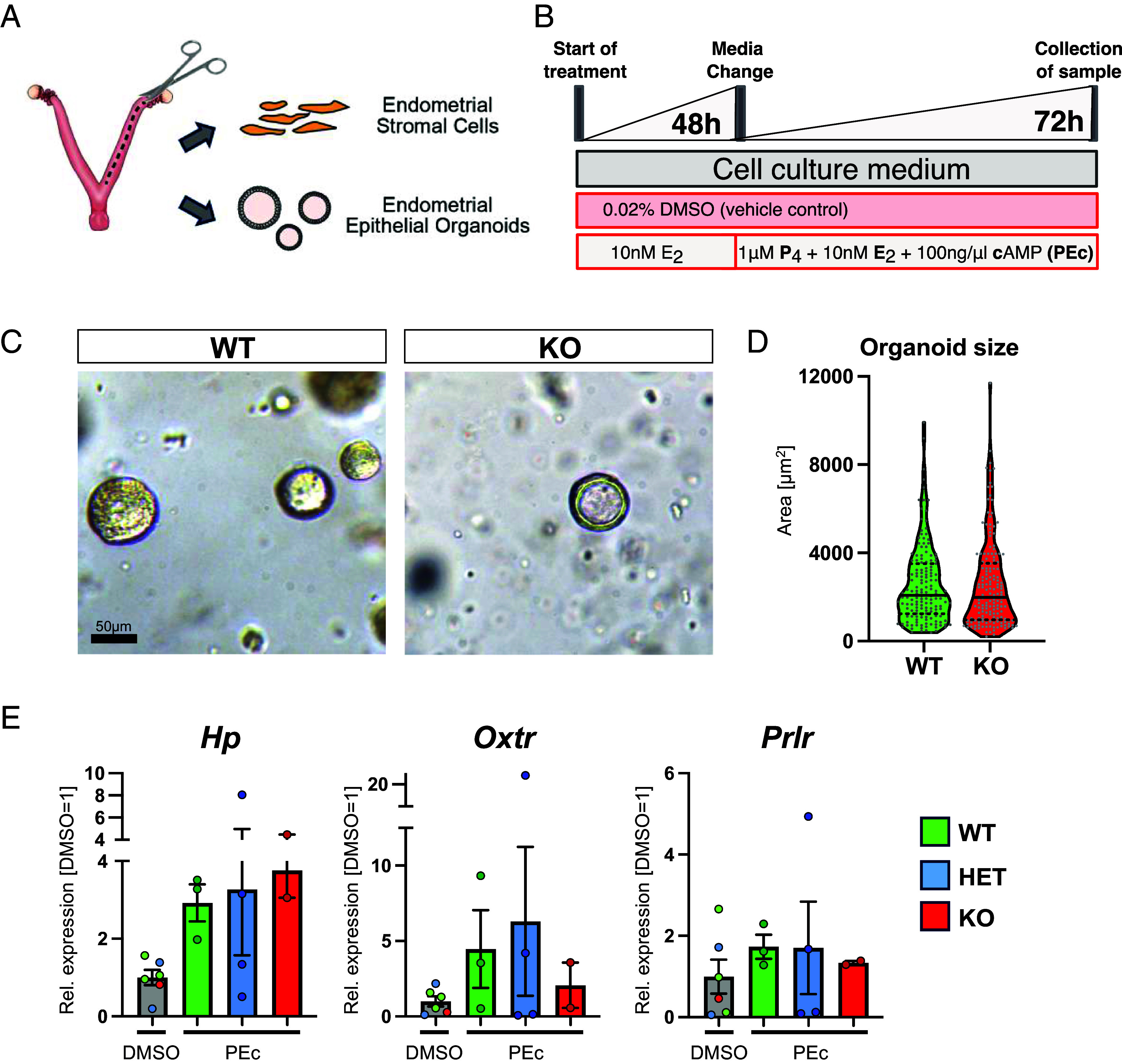
Hormonal response of epithelial and stromal cells in vitro is unaltered. (*A*) Diagram of experimental strategy for endometrial epithelial and stromal cell isolation. (*B*) Hormonal stimulation protocol in which cells were primed with estradiol for 2 d and then exposed to a cocktail of progesterone, estradiol and cAMP (“PEc”) for another 3 d. DMSO was used as vehicle control. (*C*) Images of endometrial epithelial organoids derived from WT and KO females. (*D*) Organoid size measurements after 7 d in culture. Data are from 158 WT and 163 KO individual organoids derived from n = 3 independent females each. (*E*) RT-qPCR analysis on epithelial organoids assessing expression of genes found previously to be hormonally responsive. Data points represent biological replicates (i.e., different females). Data are normalized to expression levels in vehicle-control (DMSO) conditions and are plotted as mean ± SEM. Statistical significance was calculated using one-way ANOVA with Dunnett’s multiple comparisons test.

The ability to derive endometrial epithelial organoids afforded a great opportunity to examine the characteristics of the epithelial compartment of *Atp11a* KO uteri in more detail. Alongside, we also isolated endometrial stromal cells from the same uteri and subjected both cell types to a hormonal induction protocol (Fig. 4 *A* and *B*). Despite the reduced expression of GE progenitor markers in vivo, we did not observe overt growth differences of epithelial cells, although the KO organoids tended to be smaller (Fig. 4 *C* and *D*). Expression analysis of genes selected because they failed to be hormonally induced in vivo (Fig. 3*A* and *SI Appendix*, Fig. S3*E*) showed no global differences between WT and KO organoids (Fig. 4*E*). Similarly, no consistent deficits were observed for hormone-responsive stromal cell decidualization markers in KO cells (*SI Appendix*, Fig. S5 *A* and *B*). This latter finding corroborates the histological appearance of successful implantation sites (Fig. 1*D*) in HET females as well as the lack of misregulation of early decidualization markers in E3.5 uteri of KO uteri in vivo (*SI Appendix*, Fig. S5*C*). In general, the cell culture experiments were characterized by a large degree of variability between independent biological replicates, and our replicate numbers were constrained by the extreme difficulty to obtain *Atp11a* KO females, which requires a placental rescue strategy (Mendelian ratio for obtaining these females is 1/16). Nevertheless, despite these caveats, it appears that both epithelial and stromal KO cells behaved normally when taken out of the tissue context of the uterine environment and upon exposure to identical hormone concentrations.

### Luminal Epithelial Defects in *Atp11a* KO Uteri.

**Fig. 5.**
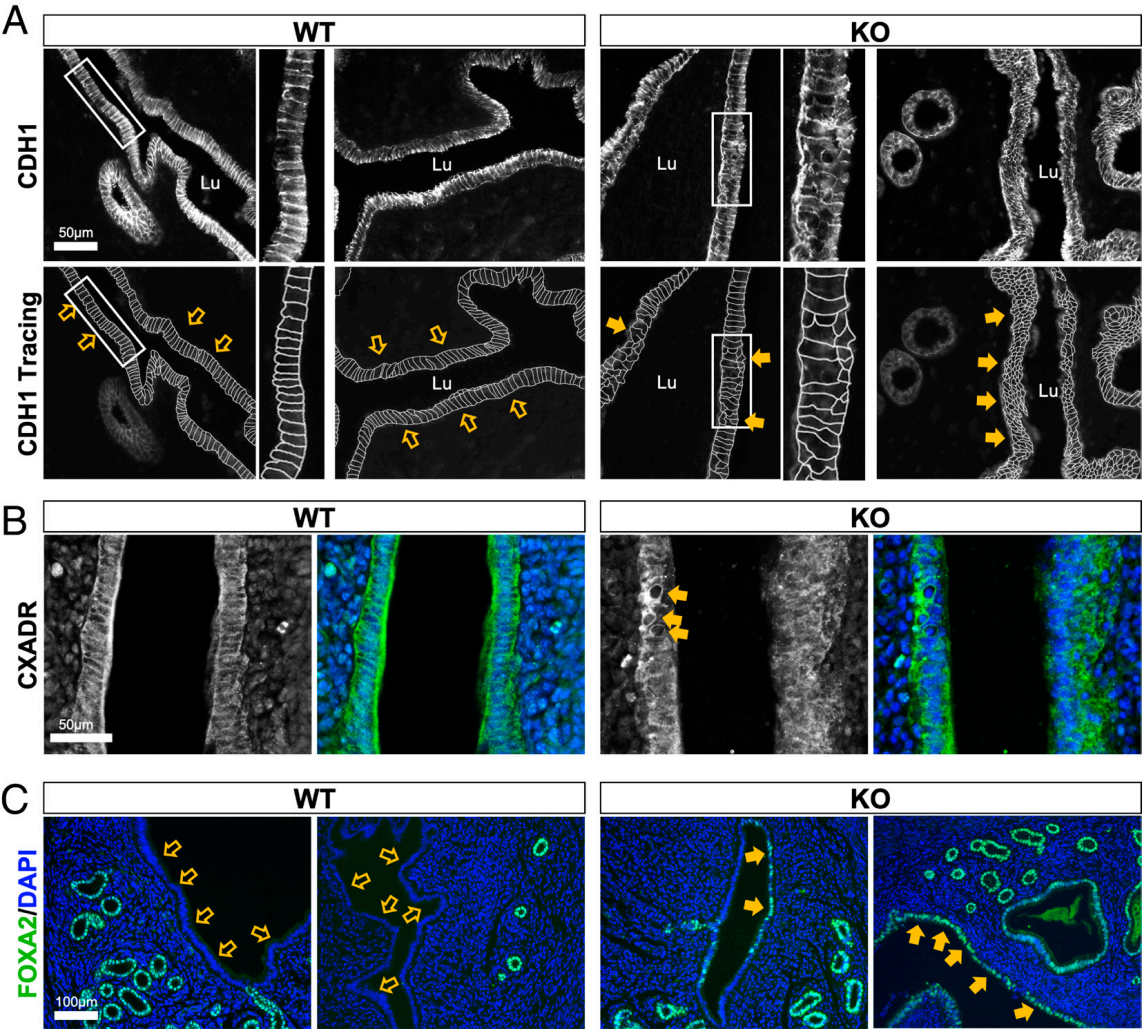
Luminal epithelial defects in *Atp11a* KO uteri. (*A*) Immunofluorescence staining of WT and *Atp11a* KO uteri for the epithelial marker E-Cadherin (CDH1) and manual tracing of CDH1 signals in the *Lower* row. Arrows point to the luminal epithelium (LE); filled arrows in the KO point to areas where the columnar epithelium of the LE appears highly disorganized with rounded cells and a multilayered appearance. The white rectangle highlights areas for which magnifications are shown, adjacent to the main image. The images are representative of n = 5 WT and KO uteri each. Lu = uterine lumen. (*B*) Staining for the tight junction protein CXADR highlights the perturbed integrity of the LE with a lack of clear cellular outlines and the appearance of vacuolar structures in the LE of KO uteri (arrows). (*C*) Immunofluorescence staining for the normally GE-restricted transcription factor FOXA2. Open arrows highlight the lack of expression of FOXA2 in the LE of WT uteri, and filled arrows highlight the ectopic expression of FOXA2 in the LE of *Atp11a* KO uteri. These cells also appear particularly rounded in morphology.

Prompted by the loss of tight junction marker CXADR in GE cells (Fig. 3*G*), we investigated the epithelial structural abnormalities in greater detail in the LE of *Atp11a* null uteri. E-Cadherin (CDH1) staining revealed frequent patches of a disrupted epithelial architecture of the columnar LE, with cells displaying a more rounded shape and often multilayered organization (Fig. 5*A*). This phenotype was even more obvious when manually tracing the CDH1 signals (Fig. 5*A*). Since these changes are reminiscent of an epithelial–mesenchymal transition phenotype, we also stained for the squamous epithelial marker p63 (35) but found no expression in KO LE cells (*SI Appendix*, Fig. S6*A*). However, the disorganization of the KO LE was particularly striking on examination of the tight junction marker CXADR. In WT uteri, the CXADR signal closely followed the cell membrane outlines, similar to CDH1 (Fig. 5*B*). By contrast, in KO uteri, CXADR staining was discontinuous and crisscrossed the LE layer, with no sharp cell boundaries in evidence (Fig. 5*B*). The CXADR staining also highlighted some abnormal vacuolar structures inside the LE, reminiscent of our previous observations in *Atp11a* KO placentas (18). This visibly disrupted architecture of the LE was further corroborated by the downregulation of various other factors, such as the chloride channel *Clcn2*, the transcription factor *Hnf1b,* and the Vitamin D receptor (*Vdr*), that are involved in tight and adherens junction formation, in epithelial integrity and in uterine receptivity (*SI Appendix*, Fig. S6*B*) (36–39).

The LE defects did not only pertain to cellular architecture but also to the expression of critical transcription factors. The reduced expression of PAX8 that we observed in the GE was also evident in the LE of KO uteri (*SI Appendix*, Fig. S6*C*). Moreover, we found that FOXA2, a normally GE-restricted factor, was misexpressed in patches of LE cells in KO but not in WT uteri (Fig. 5*C*). The FOXA2+ cells were characterized by a particularly rounded appearance. Since such LE expression is not observed in normal uteri, this ectopic expression pattern is indicative of a misspecification, or lack of specification, of *Atp11a*-null LE cells.

### Impact of Uterine Dysfunction on Placental and Fetal Development.

**Fig. 6.**
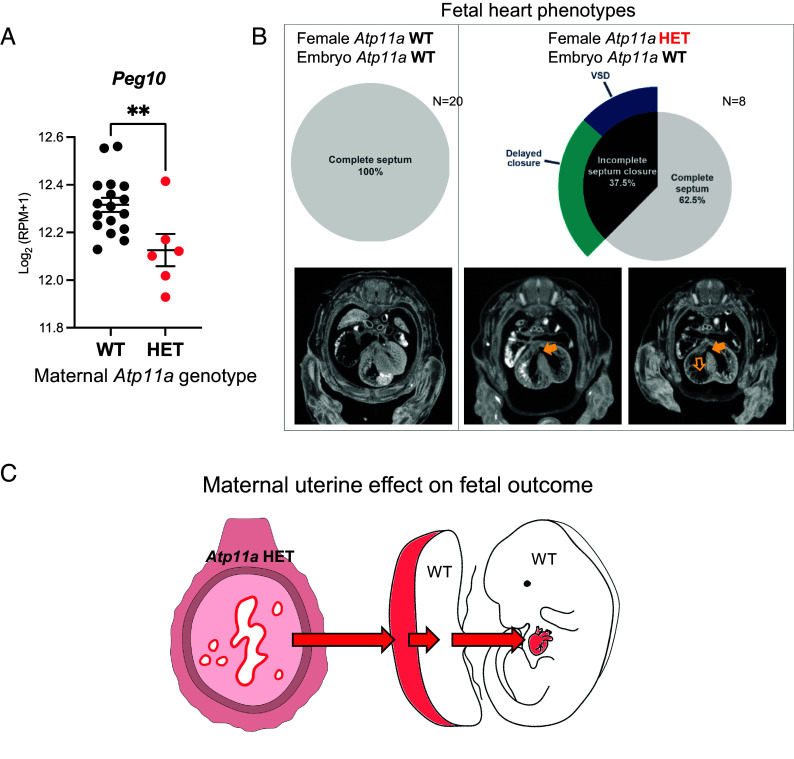
Uterine *Atp11a* deficiency causes an increased risk of placental trophoblast and fetal heart defects. (*A*) Example of a gene, *Peg10*, that is critical in placental trophoblast for normal development and that is significantly down-regulated in WT placentas to *Atp11a* HET females. For maternal genotype, “WT” refers to the *Atp11a* locus, as indeed these samples are heterozygous for the unrelated gene targeting events at the *Smg9* or *Ssr2* loci. Log_2_(RPM + 1) values are displayed as mean ± SEM. Statistical analysis was performed using the unpaired *t*-test. **P < 0.01. (*B*) Fetal heart phenotypes in WT offspring to females with an intact *Atp11a* gene locus (*Atp11a* WT) or to *Atp11a* HET females. The lack of one functional *Atp11a* allele in the mother causes an increased risk of developmental heart problems in the fetus. Filled arrows point to a mild ventricular septal defect (VSD), and the open arrow points to myocardial wall thinning in the right ventricle. µCT imaging data from ref. 18. (*C*) Diagram of the propagation of uterine defects to cause a higher risk of developmental abnormalities in the placenta and fetus.

The decidualizing uterine endometrium goes on to form an integral part of the developing mouse placenta from E10.5 onward. Remarkably, in uterus-specific *Foxa2*-deficient glandless mice in which implantation has been rescued through LIF administration, abnormalities to placental morphology have been reported (40). Therefore, we assessed whether *Atp11a* insufficiency might also have an impact on placental functionality. To this end, we reexamined our previously established RNA-seq data from E14.5 placentas of WT embryos derived from pregnancies to females heterozygous for either *Atp11a* or the unrelated genes *Ssr2* and *Smg9* which served as a control as they do not exhibit any reproductive issues (18). Using transcriptomes from separated decidual and trophoblast fractions of the placenta (41) to generate a highly trophoblast-enriched gene list, we found that the majority (n = 5/6) of WT placentas derived from *Atp11a* HET females clustered separately, away from all other samples, on a PCA plot (*SI Appendix*, Fig. S6*D*). Genes differentially expressed specifically in WT placentas of *Atp11a* HET females included trophoblast-essential genes such as *Peg10* (Fig. 6*A*) (42). Thus, *Atp11a* heterozygosity of the decidual compartment had a measurable, global impact on placental trophoblast gene expression.

Our interest in *Atp11a*, *Ssr2,* and *Smg9* was originally spurred by the fact that conceptuses mutant for these genes exhibit both placental and heart defects (21). Comprehensive conditional KO experiments further demonstrated that in case of *Atp11a* (but not for *Ssr2* or *Smg9*), the heart defects as well as the embryonic lethality were solely due to abnormalities in the placenta (18, 43). Thus, to take the current data even further, we reexamined our µCT imaging data of WT embryos from all crosses more closely and found that none of the control embryos from *Ssr2* and *Smg9* HET females (n = 20) exhibited heart defects at E14.5 (Fig. 6*B*). By contrast, 37.5% (n = 3/8) of the WT embryos to *Atp11a* HET females displayed ventricular septal closure defects (Fig. 6*B*). Two of these VSDs were classified as delayed closure as they resembled the small remaining gap in the ventricular septum that is characteristic of embryos 1 d earlier in gestation, at E13.5, and that likely closes as development progresses. Yet one WT embryo to an *Atp11a* HET mother exhibited a profound perimembranous VSD and was edematous, a common indicator of fetal heart defects (Fig. 6*B*). Thus, uterine *Atp11a* deficiency carries forward to manifest in placental abnormalities that, as a consequence, cause a considerable risk of fetal congenital heart defects even in WT conceptuses, solely as a result of maternal *Atp11a* heterozygosity (Fig. 6*C*).

## Discussion

In the current study, we followed up on our observations that far fewer litters were obtained from *Atp11a* HET females compared to females heterozygous for other, unrelated genes that we collectively investigated in the context of the placenta–heart axis (18). While the *Atp11a* HET females showed definitive signs of mating, the uteri of about half of them were merely swollen and fluid- and/or blood-filled, sometimes with the remnants of dead conceptuses. In the current study, we report that these early pregnancy losses likely arise from defects in the uterine epithelial compartment where *Atp11a* is expressed. Specifically, the compromised epithelial integrity combined with an apparent misspecification of the LE, characterized by ectopic expression of the key transcription factor FOXA2, causes complete or near-complete implantation failure in about 50% of cases. In the remainder of cases where this uterine receptivity bottleneck is overcome, females carry normal-sized litters but the embryos exhibit an increased risk of developmental heart defects. Although we cannot formally rule out maternal effects unrelated to uterine dysfunction, the profound structural and transcriptional deficits of the GE coupled to reduced GE progenitor marker expression are the most likely reasons for the observed alterations to placental gene expression that in turn feed forward to affect fetal heart development.

Interestingly, an impact of the uterine genotype on placental histopathology and/or transcriptomes has been previously reported in litters to females conditionally ablated for *Nodal* and *Foxa2*, both of which exhibit GE-restricted expression (40, 44, 45). These data laid the foundations for the concept that uterine gland activity directs placental development (40). Our findings strongly corroborate this concept. Although *Nodal* is not yet expressed in our E3.5 uteri [both in the current study and our previous data (41)] and glandular FOXA2 expression is not significantly altered in *Atp11a* KO uteri, we demonstrate rather striking reductions in glandular progenitor cell markers, notably *Sox9*, *Pax8*, *Lgr5* and *Prom1*, and a lack of response to hormonal stimulation. We show that these deficits likely induce the observed transcriptomic changes in the trophoblast portion of the placenta. Moreover, these deficits propagate to the fetus by imposing an increased risk of developmental disorders. Although the observed heart defects in our WT offspring were mild and would likely resolve during further development in most embryos, these data corroborate speculations that uterine dysfunction can indeed impact fetal development (40).

The experimental challenges imposed by the embryonic lethality of the *Atp11a* mutation prevented us from studying the reproductive behavior in complete *Atp11a* KO females in extensive detail. Thus, the low frequency of obtaining *Atp11a* KO females through placental rescue did not permit an in-depth analysis of pregnancy outcome. Yet the litters that were obtained indicated that ovulation and implantation rates were normal in those females where a successful pregnancy was established. However, pregnancy establishment was successful only about 50% of the time. This somewhat peculiar bimodal pattern is highly reminiscent of uterine-specific *Nodal* deletion that similarly results in 50% implantation failure despite normal ovulation, luteogenesis, and the production of healthy, viable preimplantation embryos (46). This phenotypic similarity is further underpinned by considerably lower expression levels of the Activin B component *Inhbb* in our *Atp11a* KO uteri (*SI Appendix*, Fig. S5*C*), supporting the notion that ATP11A function intersects with the Nodal/Activin axis in the establishment of uterine receptivity (47).

Our data are remarkable insofar as we observed this reproductive pattern even in *Atp11a* HET females. Thus, *Atp11a* appears to behave like a haploinsufficient allele with incomplete penetrance. One of the most obvious causes for such a phenotypic pattern is genomic imprinting, i.e., the monoallelic expression of a gene depending on parent-of-origin. However, we could not detect any evidence for this mode of inheritance, and neither did we observe any sex-specific effects. Rather, we found that *Atp11a* allele dosage showed a linear correlation with circulating P4 hormone levels. Since *Atp11a* itself is P4-regulated (48), this might enhance the risk of subthreshold conditions for uterine receptivity. Despite the lower circulating P4 levels, however, both HET and KO females can carry pregnancies in principle. Interestingly, we did not observe other classical signs of lack of P4 action; cell proliferation had correctly shifted from the epithelial to the stromal cell compartment in E3.5 KO uteri and, although there was increased proliferation, this was confined to the stromal cell compartment. As such, the *Atp11a*-depleted mice do not recapitulate the hallmark features associated with lack of uterine epithelial P4-PGR action (49). *Lif* expression also remained unchanged (*SI Appendix*, Fig. S5*C*). Moreover, when taken out of their tissue context in cell and organoid culture, both endometrial stromal and epithelial cells responded largely correctly to steroid hormone stimulation, indicating that hormone sensing was unaffected.

Collectively, our data converge on the interpretation that a severely perturbed epithelial architecture of the LE combined with GE dysfunction and reduced circulating P4 levels renders females prone to implantation failure. As a phospholipid flippase, ATP11A is located in the lipid bilayer of the plasma membrane and functions to maintain the asymmetry of PtdSer (and phosphatidylethanolamine) distribution that is highly enriched on the inner leaflet (20). This asymmetric lipid composition is of critical importance for many cellular processes and affects membrane fluidity, membrane curvature, calcium channel activity, and signal transduction into the cell. Thus, an impact of ATP11A ablation on epithelial structural morphology as well as on membrane dynamics for the uptake or secretion of products is feasible. We found that *Atp11a* KO uteri suffer from a severe loss of the tight junction protein CXADR and a profound disruption of the columnar epithelial architecture. Remarkably, we also detected an ectopic expression of the hallmark GE factor FOXA2, suggesting that ATP11A-directed membrane dynamics are critical for the acquisition of proper LE identity.

Moreover, we report that loss of ATP11A has a significant impact on uterine gland progenitor cells, with a stark reduction in the expression of key transcription factors implicated in uterine gland function, such as SOX9 and PAX8. Other markers of GE progenitors were equally affected, including the WNT-regulated stem cell markers *Lgr5* and *Prom1*. Thus, it is possible that the transmission of the WNT-β-catenin pathway is disrupted in *Atp11a* null uteri, which might also explain the aberrant expression of FOXA2 as a downstream target of β-catenin signaling (50). Such a defect would be akin to the reported role of ATP11A in modulating adhesion G protein–coupled receptor signaling (51). Collectively, these changes to GE expression are indicative of a substantial loss in cellular plasticity and progenitor cell capacity in *Atp11a*-deficient uteri that will affect the functionality of the glands as well as of the LE. Since uterine glands are the source of histiotrophic nutrition for the early conceptus (52), these changes will undoubtedly affect the uterine-embryo crosstalk, thus further exacerbating the increased risk of early pregnancy losses and of placental defects in *Atp11a*-depleted females.

Collectively, our data on *Atp11a* mutant females demonstrate the importance of cell membrane biology and lipid bilayer composition for uterine function, an aspect that has gained little attention in the context of female reproduction to date. Perhaps most importantly, however, our data demonstrate that maternal (uterine) defects propagate via the placenta to impose an increased risk of developmental disorders in the embryo even in the absence of any genetic abnormalities in the embryo itself.

## Materials and Methods

### Mice.

All animal work was conducted with approval by the University of Calgary’s animal care committee, and with appropriate Health Sciences Animal Care Committee (HSACC)-approved animal use protocols in place (protocol numbers AC22-0147 and AC22-0118). Mice were housed in IVC cages with 12 h light–dark cycles under ambient temperature (~22 °C) and humidity conditions. Female mice (WT C57BL/6N, HET *Atp11a*^tm1a/+^, or KO *Atp11a*^tm1d/tm1b^; see *SI Appendix*) were set up for timed matings with C57BL/6N stud males that had successfully produced litters before. The appearance of a vaginal plug was counted as embryonic day (E) 0.5. Females were dissected either on E6.5 or on E14.5, as indicated, and assessed for pregnancy. Tissues were fixed in 4% paraformaldehyde (PFA); E6.5 implantation sites and ovaries were equilibrated in 15% and 30% sucrose solutions, embedded in Clear Frozen Tissue Section Compound (VWR 95057-838), and sectioned at 5 to 10 µm on a Leica CM3050S cryostat. For ovary analysis, consecutive sections from the center of the ovaries were selected and stained for H&E. Only secondary follicles, antral follicles, and corpora lutea were included in the analysis. Each type was counted across at least 200 μm of tissue for each ovary. Fixed E14.5 embryos were transferred into phosphate-buffered saline (PBS) for imaging and crown-rump length measurements were performed in ImageJ (v1.53). Yolk sacs were used for genotyping. Genotyping primers are provided in *SI Appendix*, Table S1. Embryonic heart defects were assessed by µCT image analysis as described (18). Briefly, PFA-fixed E14.5 embryos stained with Lugol’s iodine (2.5% w/v I_2_Kl) dye overnight. Stained samples were then embedded in 1% agarose and images were taken with a ZEISS Xradia Versa 520 X-ray microscope 186 (Carl Zeiss AG, Oberkochen, Germany) (×0.4 objective, 50kV, 4W, binning 2, and exposure of 2 s). Image viewing and analysis was performed with the ZEISS XMReconstructor, ZEISS XMcontroller, and Slicer (version 4.11.20210226) software.

### Plasma Hormone Analysis.

Maternal plasma was collected at dissection through heparin injection and intracardiac blood collection. Blood was spun at 1,600 g for 10 min and then 12,500 g for 10 min and stored at −80 °C until use. Steroid/Thyroid 6-plex Discovery Assay® Multispecies Array for Serum/Plasma Samples (STTHD-Serum/Plasma) was run in duplicate on the Luminex® 200™ platform (Millipore 41116133) by Eve Technologies (Calgary, AB).

### Uterine Collection, RNA Extraction, and Tissue Processing.

Females plugged by vasectomized CD1 males were dissected at E3.5; each uterine horn was cut into two to three pieces, and one piece from each uterine horn snap-frozen in liquid nitrogen for RNA extraction, while the other pieces were fixed in 4% PFA overnight at 4 °C for paraffin histology. For RNA extraction, snap-frozen tissues were immersed in 1 mL TRI reagent (Sigma T9424) and the tissue was immediately macerated with a tissue homogenizer. RNA was isolated according to the manufacturer’s protocol. RNA quality was assessed by gel electrophoresis and on a Tapestation.

### In Situ Hybridization.

For detection of *Atp11a* transcripts, E3.5 cryosections were hybridized with an antisense and sense probe designed against a unique region of the main Atp11a transcript. In situ hybridization was performed using a standard protocol and signals detected with a fluorescein-coupled antibody (53).

### RNA Sequencing.

RNA sequencing was performed by UCalgary Centre for Health Genomics and Informatics, using the NEB Ultra II Directional RNA Library Prep kit and sequencing on an Illumina NovaSeq 6,000 (50 bp paired-end). Read pairs were aligned with STAR (2.6.1a_08-27) to the reference mouse genome (GRCm38/mm10). Count tables were assembled with htseq-count (bioconda 2018.11) using the reverse strand and nonunanimous settings, resulting in 40M to 89M gene counts per sample. All primary sequencing data have been deposited in the Gene Expression Omnibus (GEO) repository under accession GSE278900 (54). Further analyses were performed using the SeqMonk program v1.48.1 (https://www.bioinformatics.babraham.ac.uk/projects/seqmonk/) with integrated R codes for DEseq2 analysis (https://www.R-project.org/). Differential expression was calculated using DESeq2 and adjusted for multiple testing correction using the Benjamini–Hochberg method (*Padj* < 0.05). Most graphs including heatmaps, PCAs, and StarWars plots were generated in SeqMonk. Read counts per million (RPM) were calculated normalized to library size and with merged transcript isoform settings; to avoid negative values after logarithmic transformation, a numerical value of 1 was added to each gene count prior to Log_2_-transformation to generate Log_2_(RPM + 1) values. Expression values for individual genes were plotted in GraphPad Prism v10. Gene enrichment analyses were performed using Metascape (23).

Trophoblast-specific gene lists were generated from E11.5 decidual and trophoblast transcriptomes, selecting for genes exhibiting ≥fourfold higher expression in the trophoblast portion.

### Cell-Type-Specific Deconvolution Analysis.

We created a single-cell RNA (scRNA) reference dataset of mouse uterus using publicly available scRNA-seq data (Arrayexpress accession numbers: E-MTAB-11491 and E-MTAB-12889) (29). After quality checks, we merged data from different age groups and estrus cycle stages with the R package Seurat (v 5.1.0) (55). We used a sketching method implemented in Seurat to ensure equal representation of cells (n = 6,500) from each group, which samples scRNA while preserving rare cell types (56). We annotated four major clusters in our integrated reference dataset—epithelial, endothelial, stromal, and immune cells. The immune cells were further subclassified based on the expression of canonical markers. This reference dataset was utilized to perform reference-based deconvolution analysis using Bisque (v 1.0.5) (30). To extract cell-type-specific markers for deconvolution, we used the Mean Ratio method from the R package DeconvoBuddies (v 0.99.1). All scripts used for the deconvolution analysis are available at https://github.com/ankita86/Hemberger_Lab/tree/main.

### Immunofluorescence and Immunohistochemical Staining.

PFA-fixed tissues were embedded for paraffin histology and sectioned at 7 µm on a Leica paraffin microtome. Following deparaffinization and rehydration, antigen retrieval was performed by boiling in 10 mM NaCitrate pH 5.2 buffer. For p63 detection, an additional quenching step of endogenous peroxidase was performed with 3% hydrogen peroxide in PBS. Immunostainings were performed using standard procedures. For details, please refer to *SI Appendix*.

For H&E staining, paraffine sections were deparaffinized and rehydrated, and cryosections were dried and rehydrated, before subjecting them to a standard histological staining protocol using Harris’ modified Hematoxylin solution (Sigma HHS32) and alcoholic Eosin Y solution (Sigma HT110116).

### Endometrial Epithelial and Stromal Cell Culture.

Endometrial epithelial and stromal cells were isolated following previously established (28, 41). Endometrial epithelial organoids were cultured in phenol red-free DMEM/F12 supplemented with a cocktail of signaling and growth factors (28). Organoids were used at first passage for all experiments. Stromal cells were cultured in phenol red-free DMEM/F12 medium (Wisent 319-080 CL) supplemented with 10% fetal bovine serum (FBS, Wisent 098150) and 1% penicillin/streptomycin (Wisent 450115-CL) For hormone treatment, cells were exposed to 10 nM estradiol (Sigma E2758) for 48 h, followed by exposure to 10 nM estradiol, 1 μM progesterone (Sigma P8783), and 100 ng/μL cAMP (Sigma B5386) for 72 h. Vehicle controls [0.02% DMSO (Thermo Fisher J66650-AD)] were included for each biological replicate. See also *SI Appendix*.

### RNA Isolation and RT-qPCR Analysis.

Total RNA was extracted using the Qiagen RNeasy Mini kit (Qiagen 74104). 100 to 1,000 ng of total RNA was used for cDNA synthesis with RevertAid H-Minus reverse transcriptase (Thermo Scientific EP0451). Quantitative (q)PCR was performed using SsoAdvanced SYBR Green Supermix (BioRad 1725274) and intron-spanning primer pairs on a Bio-Rad CFX96 or CFX384 thermocycler. Expression values were normalized to the housekeeping gene *Sdha* and are displayed relative to vehicle-control (DMSO) conditions. Error bars indicate SEM of independent replicates, with individual data points displayed in the graphs. PCR primers are provided in *SI Appendix*, Table S1. Data were statistically analyzed using GraphPad Prism v10.4.1.

## Supplementary Material

Appendix 01 (PDF)

## Data Availability

All primary sequencing data have been deposited in the GEO repository under accession number GSE278900 (54). Single-cell deconvolution code is available at https://github.com/ankita86/Hemberger_Lab/tree/main (57).
