# Supplementary material for: Phospholipid flippase ATP11A brokers uterine epithelial integrity and function
Source: Proc Natl Acad Sci U S A. 2025 Apr 22;122(17):e2420617122. doi: 10.1073/pnas.2420617122 (PMC12054786; doi:10.1073/pnas.2420617122)
Supplement: Supplementary file 1 — Appendix 01 (PDF) [file pnas.2420617122.sapp.pdf]

Supporting Information

Phospholipid flippase ATP11A brokers uterine epithelial integrity and function

Alexa Krala[1,2], Aleksandra O. Tsolova[1,2], Bethany N. Radford[1,2], Anshul S. Jadli[1,2], Xiang Zhao[2,3], Danielle Blackwell[1,2], Ankita Narang[2], Wendy Dean[2,3] and Myriam Hemberger[1,2,*]

Suppl. Figure 1

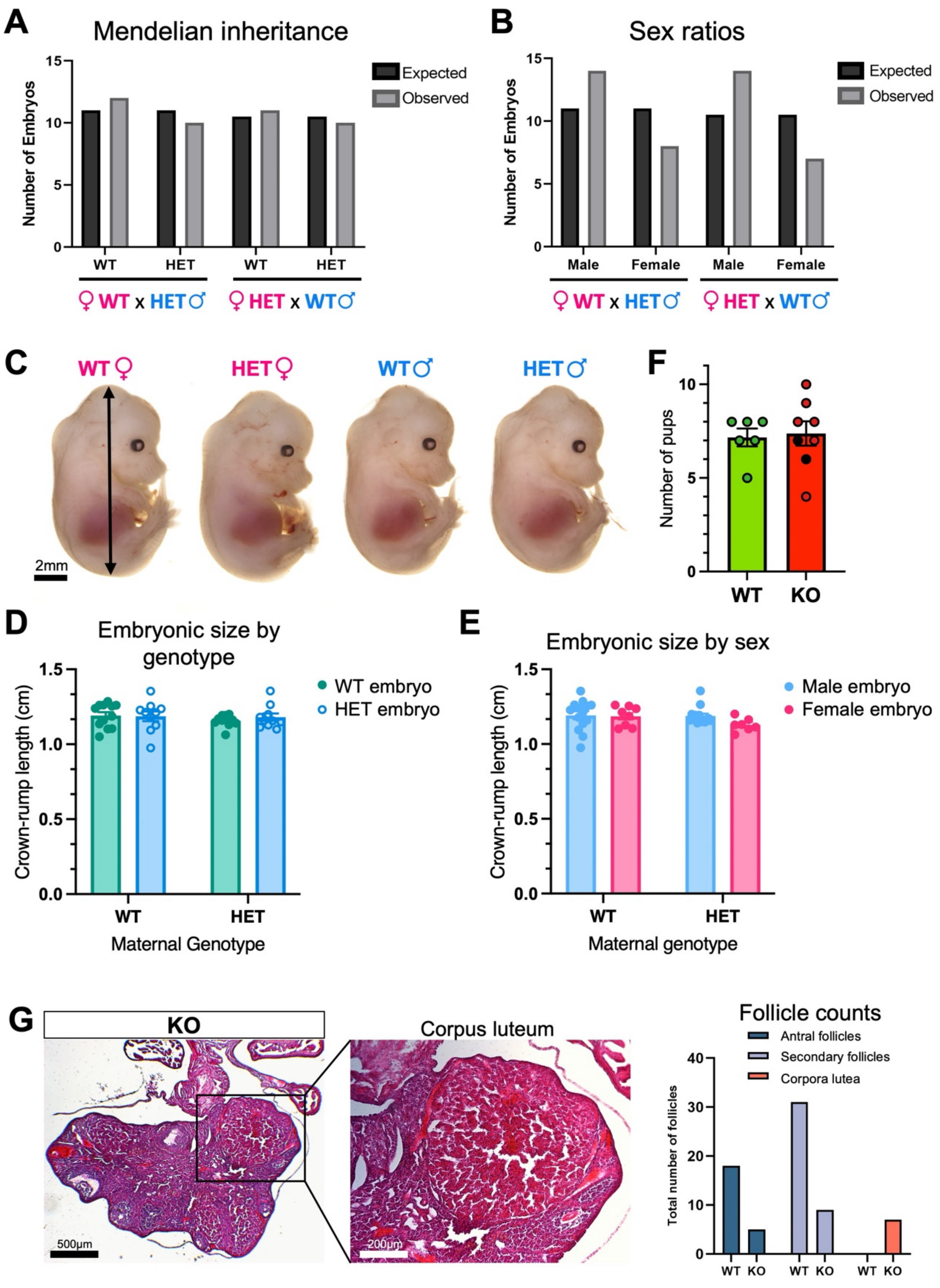

Suppl. Figure 1. Lack of genomic imprinting, sex-specific effects and ovulation defects.

(**A**) Number of wild-type (WT) and heterozygous (HET) embryos developed in reciprocal crosses between WT and *Atp11a* HET parents. If *Atp11a* was subject to genomic imprinting, the HET offspring should be depleted in one type of these crosses specifically. (**B**) Sex ratios of embryos obtained from these crosses. (**C**) Examples of WT and HET embryos (female and male examples shown for each) on which crown-rump length measurements were performed. (**D**) Crown-rump length measurements displayed as a function of embryo genotype. Data are shown as mean +/- S.E.M. (**E**) Crown-rump length measurements displayed as a function of embryo sex. Data are shown as mean +/- S.E.M. (**F**) Litter sizes of offspring obtained to WT and *Atp11a* KO females at weaning. Black datapoints indicate litters in which all pups died before weaning. (**G**) Analysis of adequate follicle maturation and corpus luteum formation in ovaries of HET and KO females.

Suppl. Figure 2

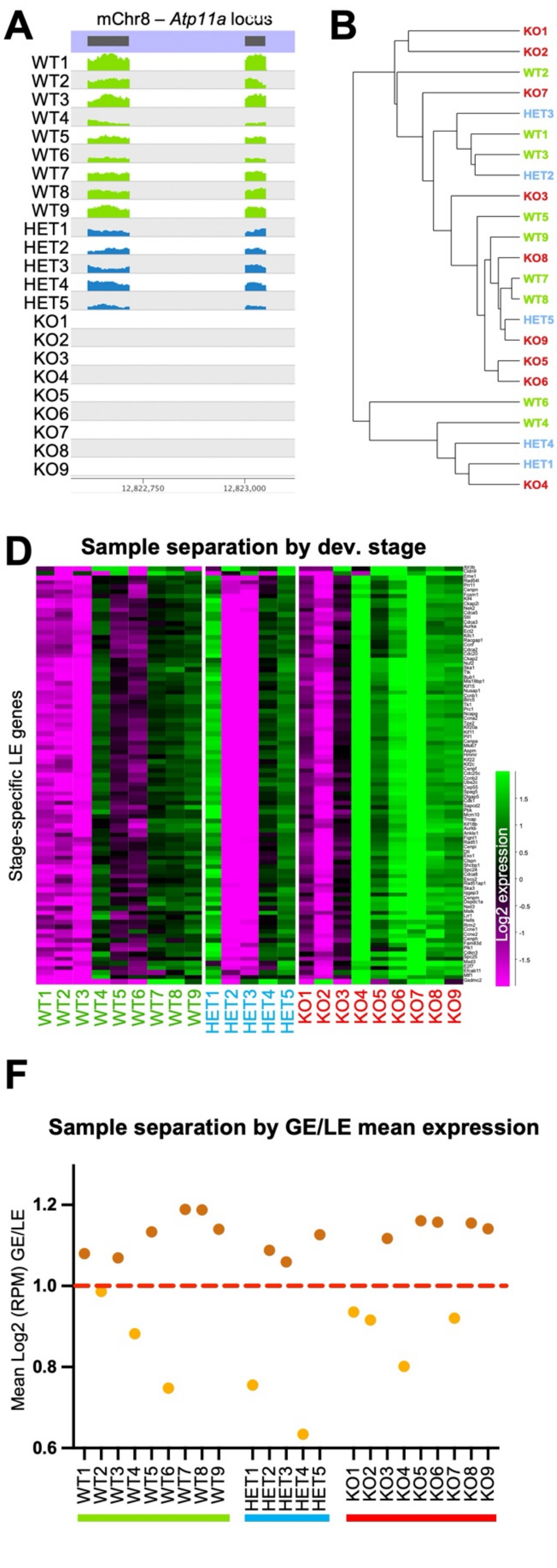

Suppl. Figure 2. Informed stratification of uterine RNA-seq samples.

(**A**) Wiggle plot across *Atp11a* exons 7 and 8 that are correctly deleted in the KO samples. (**B**) Tree diagram based on Euclidean distances demonstrating a lack of sample clustering by genotype. (**C**) PCA graph on the entire transcriptomes of all samples. (**D**) Heatmap of stage-specific LE genes (Ref. 22) indicates sample separation by precise developmental stage. (**E**) Highlighting the identified E3.5-early and E3.5-late samples on the PCA graph of all genes (same as in (C)) shows that largely, the precise developmental stage separates the samples along PC2. (**F**) Ratios of mean GE and LE expression values from E3.5 laser-microdissected tissue (Ref. 7) highlights some samples with higher and lower GE/LE ratios. (**G**) Highlighting the "GE-high" and "LE-high" samples from (F) on the PCA of all genes (same as in (C)) separates the samples along PC1. Of note, the GE-high samples do not contain visibly different numbers of glands (see Fig. 3D and Suppl. Fig. 3E, 4A). This stratification of data was used for the supervised selection of samples (red rectangle) shown in Fig. 2B that exhibit the most homogenous expression profiles overall. Importantly, the critical differences that we observed in our stage-matched subgroups, such as the reduced abundance of PAX8 and SOX9 in GE and the loss of epithelial integrity in LE, were also present in the remainder of the KO samples.

Suppl. Figure 3

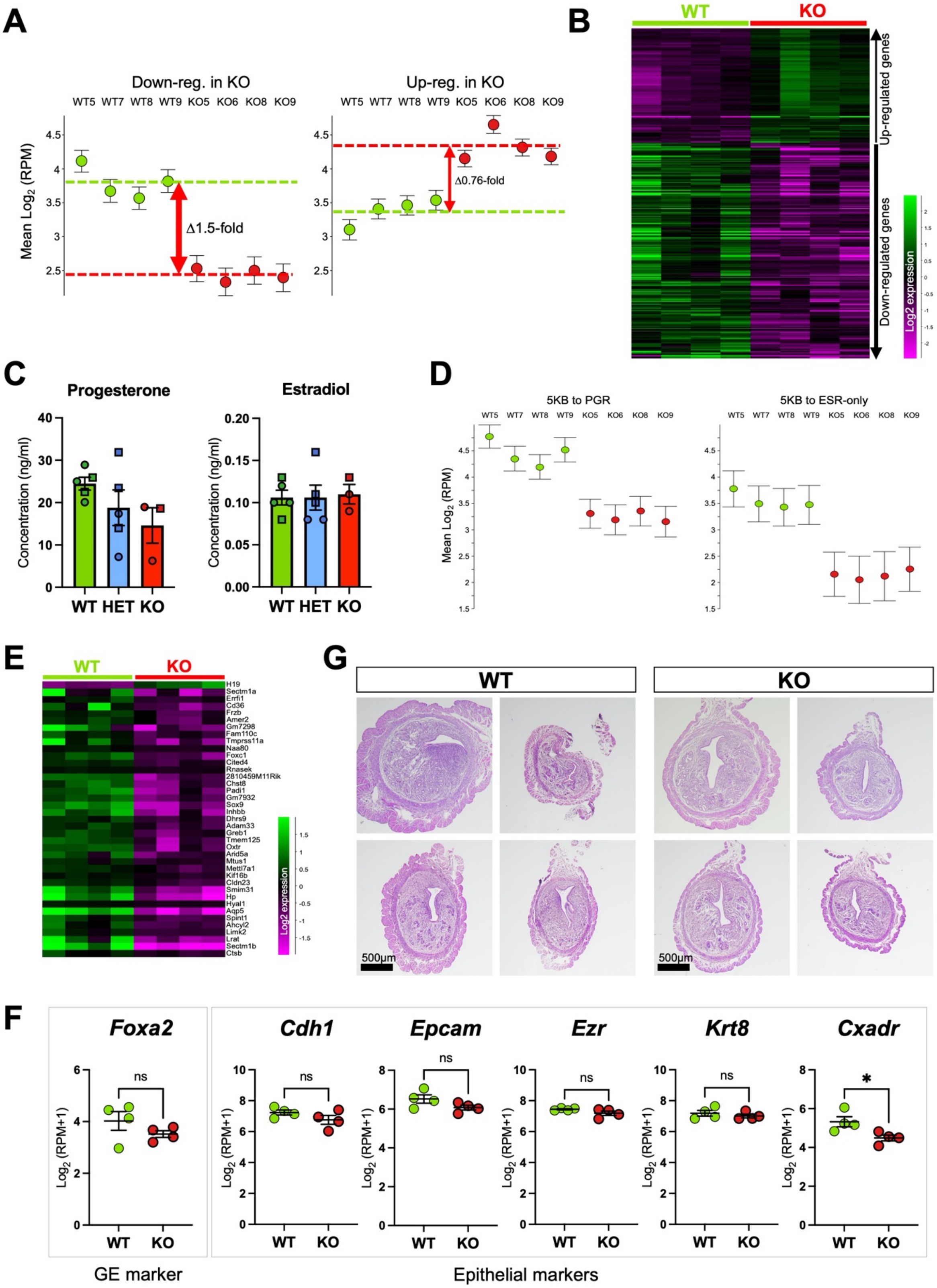

Suppl. Figure 3. Further characterization of selected samples.

(**A**) “StarWars” plots displaying mean expression levels of differentially expressed up- and down-regulated genes, colour-coded for genotype. The extent of down-regulation is far more pronounced than the extent of up-regulation, suggesting a greater impact of the down-regulated gene cohort. (**B**) Heatmap of differentially expressed (DE) genes between the selected WT and KO samples (n=4 each) demonstrates the more severe transcriptional differences for the down-regulated genes compared to the up-regulated ones. (**C**) ELISA for circulating progesterone and estradiol levels in blood plasma of non-pregnant (circle) or E6.5 pregnant (square) WT, HET and KO females. Pregnancy status did not have any major impact on values obtained. (**D**) “StarWars” plots displaying mean expression levels of genes that are down-regulated in KO uteri and close to (within 5kb) progesterone receptor (PGR) or estrogen receptor (ESR) binding sites. (**E**) Heatmap of genes that are strongly hormonally regulated in WT endometrial gland organoids and also mis-expressed in *Atp11a* KO uteri. Except for *H19*, all of these genes are expressed at far lower levels in KO compared to WT uteri, suggesting a lack of proper activation. (**F**) Expression values based on RNA-seq of generic epithelial markers such as *Cdh1*, *Epcam*, *Krt8* and microvillous marker *Ezr* are unchanged between WT and KO uteri. However, mRNA expression of the tight junction marker *Cxadr* is down-regulated in KO samples, in line with the severely depleted protein levels shown. Log_2(RPM+1) values are displayed as mean +/- S.E.M. Statistical analysis was performed using two-tailed unpaired t-test. *$p<0.05$. (**G**) H&E staining of uterine sections of all 4 WT and 4 KO samples selected for RNA-seq analysis.

Suppl. Figure 4

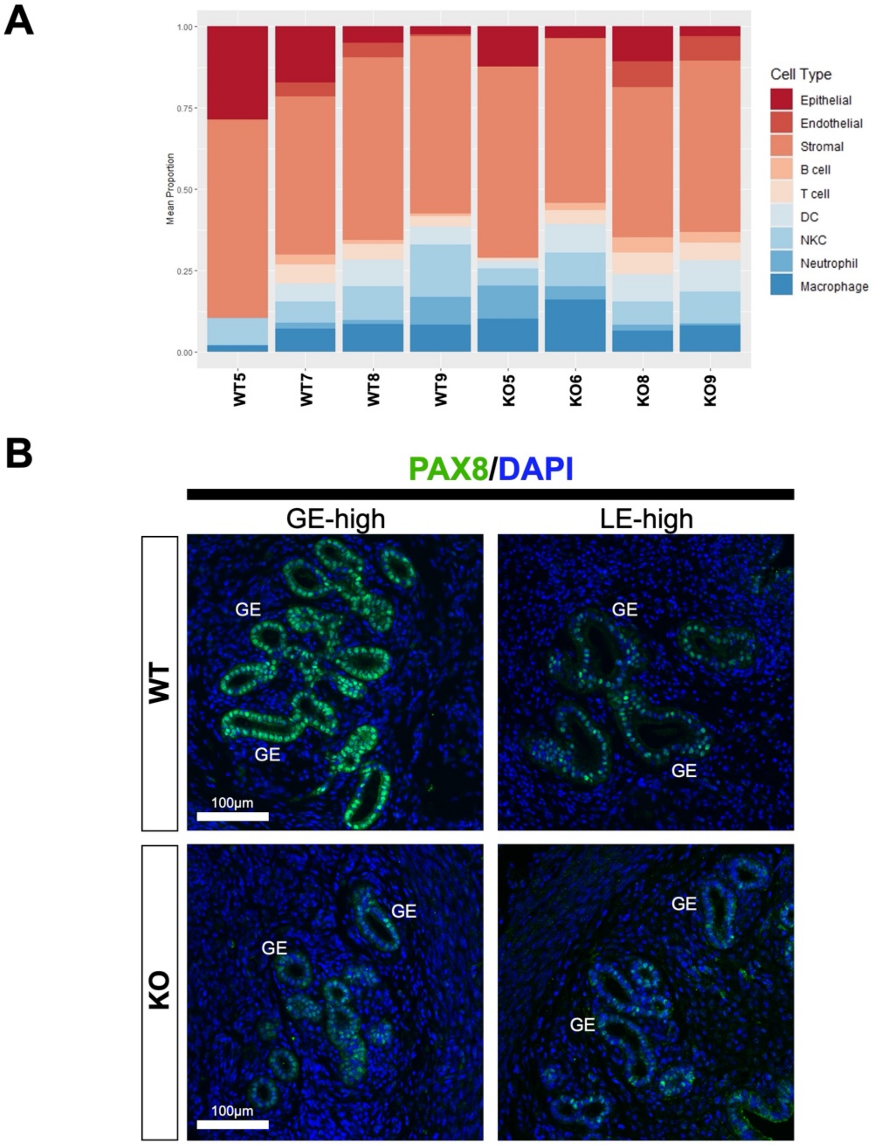

Suppl. Figure 4. Glandular defects in *Atp11a* KO uteri.

(**A**) Estimated cell proportions determined by bioinformatic deconvolution of our bulk RNA-seq data based on single cell sequencing information (Ref. 33) using bisque analysis (Ref. 32). (**B**) Immunofluorescence staining for the glandular epithelial progenitor marker PAX8. PAX8 staining intensity is dramatically reduced in the selected GE-high KO samples. Although overall expression is lower, a proportional reduction can similarly be made out in the LE-high samples, demonstrating the validity of the results across the entire set of samples.

Suppl. Figure 5

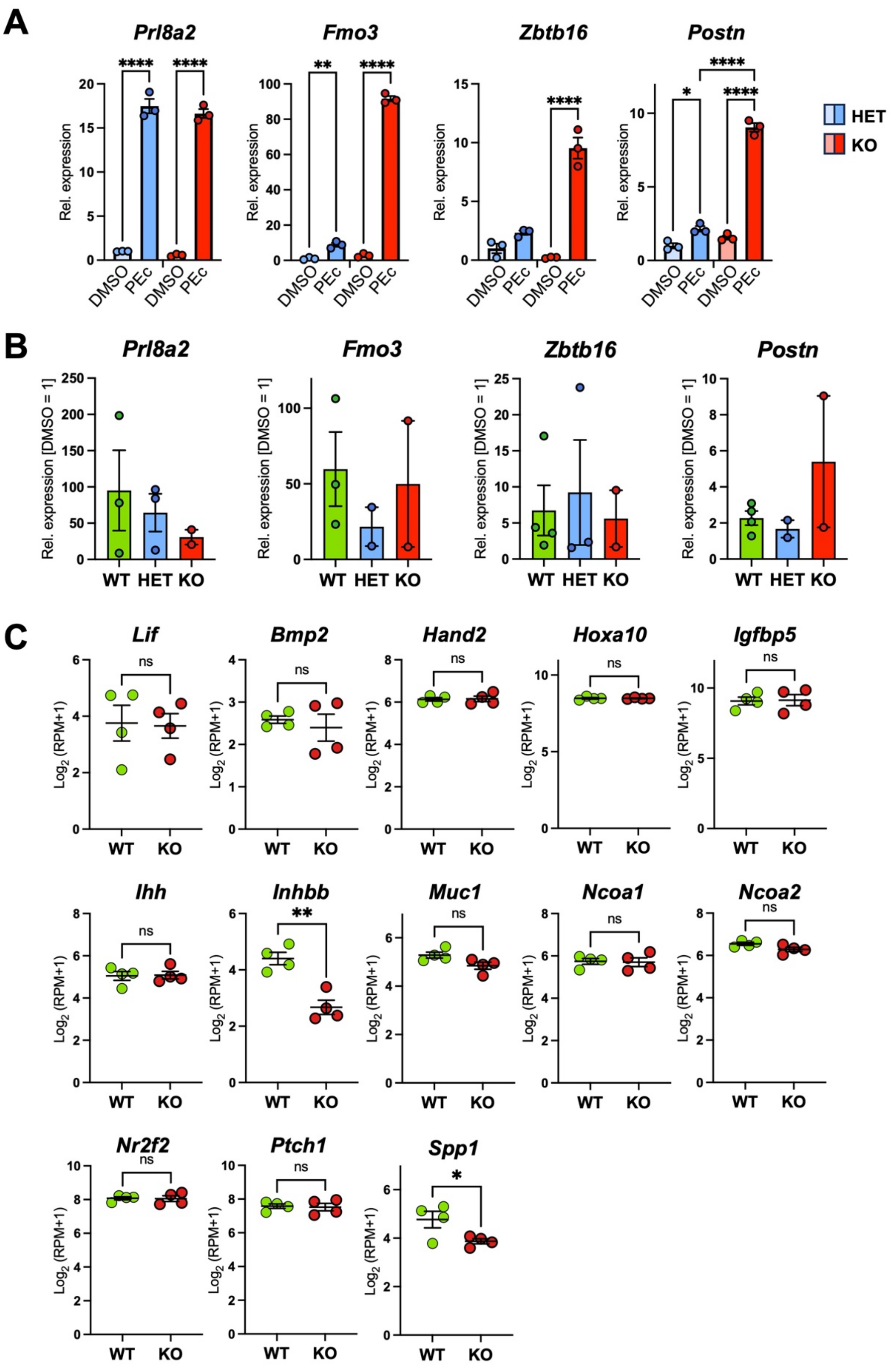

Suppl. Figure 5. Decidualization markers remain largely unaffected in *Atp11a* KO uteri.

(**A**) RT-qPCR analysis of vehicle-control and hormone-treated stromal cells for four strongly hormonally-induced genes, confirming proper activation of cells. Data points are technical replicates from one female each, and are displayed as mean +/- S.E.M. Statistical analysis was performed using one-way ANOVA. *p<0.05; **p<0.01; ****p<0.0001. (**B**) RT-qPCR expression analysis of hormonally-activated genes in stromal cells. Data are normalized to expression levels in vehicle-control (DMSO) conditions and are plotted as mean +/- S.E.M. (**C**) Expression of a cohort of common decidualization markers that largely remain unchanged in E3.5 *Atp11a* KO uteri. Exceptions are the Activin B-encoding component *Inhbb* and osteopontin, encoded by the *Spp1* gene, a membrane-tethered and integrin-binding protein, that are both reduced in *Atp11a* KO samples. Log_2(RPM+1) values are displayed as mean +/- S.E.M. Statistical analysis was performed using two-tailed unpaired t-test. *p<0.05; **p<0.01.

Suppl. Figure 6

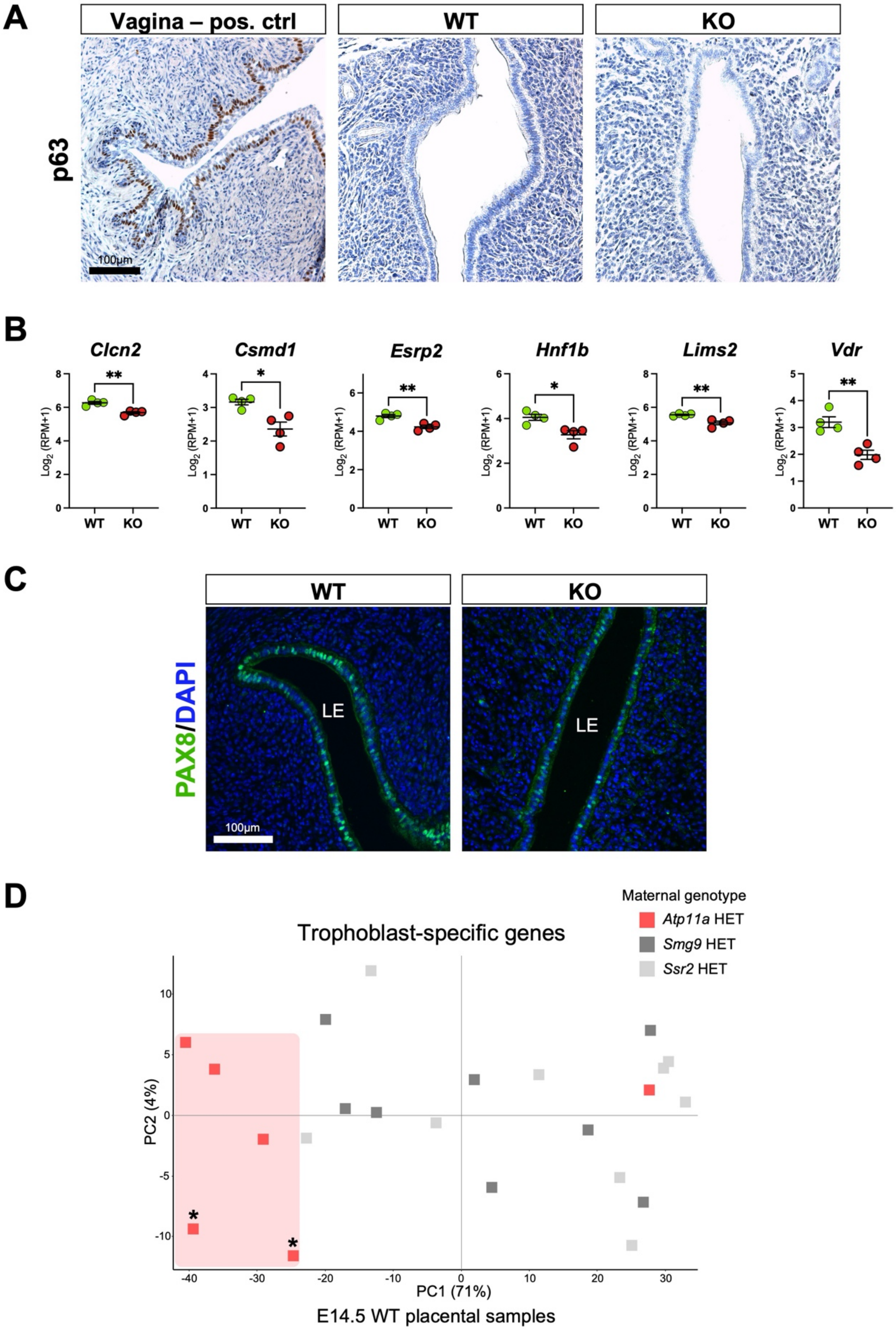

Suppl. Figure 6. Luminal epithelial defects in *Atp11a* KO uteri.

(**A**) p63 immunohistochemical staining on vagina as positive control, and on *Atp11a* WT and KO uteri. Despite morphological changes in the LE of KO females, no p63 expression was detected. (**B**) Immunofluorescence staining for PAX8 shows reduced expression in the LE of *Atp11a* KO samples. (**C**) Expression of markers of epithelial integrity, quantified as Log_2(RPM+1) from the RNA-seq data and displayed as mean +/- S.E.M. Statistical analysis was performed using unpaired t-test. *p<0.05; **p<0.01. (**D**) Principal component analysis using highly trophoblast-enriched genes on E14.5 WT placentas developed in females heterozygous for *Atp11a*, *Smg9* or *Ssr2*. In 5/6 placentas to *Atp11a* HET mothers, the trophoblast expression profile causes the samples to cluster away from the control samples that do not exhibit any reproductive issues. Placental samples marked with an asterisk demarcate those in which the fetus also exhibited a heart defect.

Supplementary Materials and Methods

Mice

The *Atp11a* gene targeted mouse line was purchased from the International Mouse Phenotyping Consortium (IMPC), and derived and maintained as described in Radford et al., 2023 (Ref. 18). The strain was maintained as heterozygotes by routine breeding to C57BL/6N-Elite (Charles River) mice. For generation of *Atp11a* KO females, FLP-converted floxed tm1c alleles were used and bred to tm1b/+; *Sox2*-Cre transgenic males (Ref. 18). The required *Pgk1*-FlpO line (B6.Cg-Tg(Pgk1-flpo)10Sykr/J, strain ID 011065) and the *Sox2*-Cre line (B6.Cg-Edil3<Tg(Sox2-cre)1Amc>/J, strain ID 008454) were purchased from Jackson Laboratories.

Endometrial epithelial and stromal cell isolation

Endometrial epithelial and stromal cells were isolated following previously established. Briefly, uterine horns were opened longitudinally and incubated in 2.5% pancreatin (Sigma P3292), 0.25% trypsin (Sigma T4799) solution for 1 hour. Following incubation, the horns were transferred to HBSS (Sigma H4641) and, under a dissection microscope, the uterine epithelium was mechanically separated from the uterine tube using fine forceps. The epithelial sheets were homogenized in HBSS to create a uniform cell suspension. Epithelial cells were resuspended in 90% Matrigel (Corning 356231) and 10% phenol red-free DMEM/F12 (Wisent 319-080 CL), and plated in dome-shaped droplets. The organoids were cultured in phenol red-free DMEM/F12 supplemented with a cocktail of signaling and growth factors as previously described (Ref. 27); medium was changed every 2 days. Organoids were used at first passage for all experiments.

To isolate stromal cells, the uterine horns were further digested with 1 mg/mL Collagenase I (Sigma C2674), 0.05% Trypsin-EDTA (Wisent 325-043-EL) at 37°C for 30 minutes with shaking (200 rpm). After digestion, the stromal cell suspension was centrifuged at 500 × g for 7 minutes. Stromal cells were cultured in phenol red-free DMEM/F12 medium (Wisent 319-080 CL) supplemented with 10% fetal bovine serum (FBS, Wisent 098150) and 1% penicillin/streptomycin (Wisent 450115-CL) at 37°C in a 5% CO_2 incubator. Media was changed every 2 days.

Hormonal treatment regimen

For hormone treatment, cells were exposed to 10 nM estradiol (Sigma E2758) for 48hrs, followed by exposure to 10 nM estradiol, 1 µM progesterone (Sigma P8783), and 100 ng/µl cAMP (Sigma B5386) for 72 hrs. Vehicle controls (0.02% DMSO (Thermo Fisher J66650-AD)) were included for each biological replicate.

Immunofluorescence and immunohistochemical staining

PFA-fixed tissues were embedded for paraffin histology and sectioned at 7µm on a Leica paraffin microtome. Following deparaffinization and rehydration, antigen retrieval was performed by boiling in 10mM NaCitrate pH 5.2 buffer. For p63 detection, an additional quenching step of endogenous peroxidase was performed with 3% hydrogen peroxide in PBS. Blocking was performed using PBS, 0.1% Tween-20, 0.5% bovine serum albumin (PBT/BSA) for 30-60 min at room temperature. Primary antibody incubations were performed overnight at 4°C. Following washes, sections were incubated either with the corresponding AlexaFluor-conjugated secondary antibodies (ThermoFisher Scientific) diluted 1:500 in PBT/BSA, or with an appropriate biotinylated antibody at 1:200 dilution, followed by detection with AlexaFluor-conjugated Streptavidin diluted 1:500 in PBT/BSA. Counterstaining was

performed with 1µg/ml DAPI. Background fluorescence was quenched with TrueBlack Lipofuscin Autofluor Quencher (VWR 10119-144) solution. All antibody details are provided in the Supporting Information

Primary antibodies and dilutions used were as follows: CXADR 1:100 (R&D Systems AF2654); ESR1 1:400 (Cell Signaling 13258S); FOXA2 1:200 (Cell Signaling 8186); Ki67 1:150 (BD Biosciences 550609); CDH1 1:200 (BD Biosciences 610182); PAX8 1:400 (Protein Tech 10336-1-AP); PGR 1:400 (Cell Signaling 8757S); SOX9 1:100 (Abcam ab185966); p63 1:200 (New England Biolabs 39692T). For immunohistochemical detection of p63, ExtrAvidin-Peroxidase 1:100 (Sigma E2886) was carried out with the Liquid DAB+ Substrate Chromogen System (DAKO K3468). Counterstaining was performed with Haematoxylin (Sigma HHS32).

For H&E staining, paraffine sections were deparaffinized and rehydrated, and cryosections were dried and rehydrated, before subjecting them to a standard histological staining protocol using Harris' modified Haematoxylin solution (Sigma HHS32) and alcoholic Eosin Y solution (Sigma HT110116).

Supplementary Table 1: Table of primers

Experiment	Target	Primer	Sequence (5'-3')
**Mouse Genotyping primers**	Atp11a-tm1a/1c/1d	FWD	CACGTCTGTGTTCCTGTGTCC
		REV	TATTTGATGCACCTGCCCTG
		Cas1-R	TCGTGGTATCGTTATGCGCC
	tm1b-generic	FWD	CTCCCACACCTCCCCCTGAA
		REV	TGAACTGATGGCGAGCTCAGA
	Cre	FWD	CAATTTACTGACCGTACACC
		REV	TCCCCAGAAATGCCAGATTAC
	Flp	FWD	TCTTTAGCGCAAGGGGTAGGATCG
		REV	GTCCTGGCCACGGCAGAAGC
	Cts8-internal control	FWD	CAGTTTGGATTCTGAATGGC
		REV	ACAGCCTCTTTTTCTCCAGTC
	Sry	FWD	TTGTCTAGAGAGCATGGAGGGCCATGTCAA
		REV	CCACTCCTCTGTGACACTTTAGCCCTCCGA
**RT-qPCR primers**	Prlr	FWD	GTGGCCGTTCTCTCTGCTGT
		REV	TTTTGGCCCCGGAACTGGTG
	Postn	FWD	TCATTGAAGGTGGCGATGGTCA
		REV	GCCCTTGAACCCTTTTGTTGGC
	Prl8a2	FWD	CAAACCCACCAGCTCATGGAC
		REV	AGGAGTGATCCATGCACCCATAA
	Fmo3	FWD	TGGCAGTGATTGGCCTGGTC
		REV	TGTCGTTTACAGAAGGCAAAGTGC
	Zbtb16	FWD	AAGCACCAGTTGGAGACGCA
		REV	GGTGCTTGATCATGGCCGAGT
	Oxtr	FWD	TGGCCTTCATCGTGTGCTGG
		REV	CAGGTACCGAGCAGAGCAGC
	Hp	FWD	ACTCTCCTGCTCTGGGGTCA
		REV	TAGAGCCACCGATGATGCGC
	Sdha	FWD	TGGTGAGAACAAGAAGGCATCA
		REV	CGCCTACAACCACAGCATCA
